# Analyzing Non-urgent Emergency Department Visits: Patterns, Demographics, Motivations, and Triage System Awareness in Al-Qassim

**DOI:** 10.7759/cureus.58383

**Published:** 2024-04-16

**Authors:** Ali M Alhojelan, Yasser Abdalmohsen Al Rusayni, Ebrahim Alsaif, Rayan K Aldoubiab, Abdulhakeem Aloqla, Ahmed A Aloraini, Rabab Alswyan, Turki S Alharbi

**Affiliations:** 1 Emergency Medicine Department, King Fahad Specialist Hospital, Buraidah, SAU; 2 Emergency Medicine Department, Buraidah Central Hospital, Buraidah, SAU; 3 Department of Surgery, College of Medicine, Qassim University, Buraidah, SAU

**Keywords:** saudi arabia, urgent medical cases, primary healthcare (phc), non-urgent medical cases, ed triage system, emergency department(ed)

## Abstract

Objective: This cross-sectional descriptive study aimed to ascertain the prevalence of non-urgent cases, investigate gender and age preferences, and explore factors influencing individuals' selection of the emergency department (ED) for non-urgent cases in the Al-Qassim region of Saudi Arabia.

Methods: From July 15, 2022, to December 31, 2022, a structured online questionnaire via a Google Docs survey collected data. The study sampled 425 patients from two prominent Al-Qassim healthcare institutions: Buraydah King Fahad Specialized Hospital and Buraydah Central Hospital. Encompassing patients aged 20 to 80 admitted to the ED between 8:00 and 16:00, concurrent with primary healthcare center availability, the study categorized participants by urgency using the Canadian Triage and Acuity Scale (CTAS) criteria. Data analysis employed descriptive statistics, chi-square tests, and probit regression in R version 4.3.3, with a significance level of *p *< 0.05 for result interpretation.

Results: In Al-Qassim in 2022, 82.4% of ED visitors sought care for non-urgent cases, while 17.6% sought care for urgent cases. No statistically significant relationship was found between age and gender and urgent ED visits. Among those with non-urgent conditions, the top reasons for bypassing primary healthcare services were slow treatment (52.7%), lack of knowledge about primary healthcare (PHC) services (33.9%), and appointment unavailability (5.5%). Evaluation of awareness of the ED triage system showed a significant difference only among patients with excellent awareness.

Conclusion: The investigation found a higher prevalence of non-urgent cases, especially among females. Challenges in appointment booking, accessibility, and the perception of urgency were key factors leading patients to choose the emergency department over PHC centers. The study emphasized the crucial role of ED triage system awareness and the impact of satisfaction with PHC services on healthcare-seeking behavior, with younger individuals less likely to visit the ED and males more inclined at specific satisfaction levels.

## Introduction

Al-Qassim, one of Saudi Arabia's administrative regions with its central hub in Buraidah, encompasses an estimated population of 1,016,756 over an area of 65,000 square kilometers [1]. The region comprises 21 primary healthcare (PHC) facilities [2] and houses King Fahad Specialist Hospital (KFSH) in Buraidah, which is recognized as a tertiary hospital boasting 445 beds [3].

Emergency departments (EDs) play a vital role in communities by delivering urgent care to individuals with injuries or illnesses requiring immediate attention. They serve as a backup when patients cannot access certain treatments in PHC. However, prolonged wait times in the ED pose a significant challenge, correlating with higher mortality rates and increased admissions [4].

The following study had four main objectives. First, it aimed to determine the prevalence of non-urgent cases in the ED of the Al-Qassim region. Second, it sought to understand the gender and age preferences of patients who selected the ED for non-urgent medical issues. Third, the study explored the factors influencing individuals' choice of the ED for non-urgent care. Lastly, it assessed the level of patient awareness concerning the triage system during ED visits. 

Overcrowding in EDs worldwide has become a critical issue, and there is a prevalent belief that the unnecessary use of the ED is a key contributing factor [5]. As a result, it is imperative to investigate and address the underlying causes of this problem to enhance the efficiency of emergency healthcare services. Non-urgent visits to the ED occur when delaying care for several hours would not significantly increase the risk of negative outcomes [5]. Efficient resource allocation and timely care for critical patients hinge on this distinction. However, these non-urgent visits can hinder the management of life-threatening cases and lower both patient and staff satisfaction [6]. Addressing this issue is crucial for EDs to prioritize effectively and provide timely care to those in critical need.

Nevertheless, patients have been utilizing EDs for non-urgent care for decades [7]. This trend has been associated with factors, such as low socioeconomic status, the young age of patients, and a lack of education [8]. To address this issue comprehensively, it is essential to consider these underlying factors and develop effective strategies for redirecting non-urgent cases to more appropriate healthcare settings.

According to a report from the Canadian Institute for Health Information, 57% of all ED visits are for non-urgent or less urgent cases [9]. An Iranian study involving 1,884 patients found that 64.6% of patients were categorized as non-urgent cases, with 36.6% seeking rapid treatment, primarily during night shifts and weekends, and their symptoms typically lasted less than a week [10]. Similarly, a study conducted in a Turkish hospital, involving 624 patients, revealed that 227 of them visited the ED seeking quicker examination, while 193 could not secure early appointments with alternative health units. In addition, 126 patients had no specific reason; they simply preferred the ED over alternative health units [11]. These findings underscore the need for strategies to redirect non-urgent cases to more appropriate healthcare settings and alleviate the burden on EDs.

The healthcare system in Saudi Arabia has undergone significant transformation through Vision 2030 and the National Transformation Program. These initiatives are committed to enhancing primary care as a critical element in improving access and availability [12]. To enhance the healthcare system in Saudi Arabia, the Ministry of Health (MOH) introduced a referral system with the goal of providing specialized care for specific cases. This system also outlines that referrals to the EDs are reserved for situations that cannot be managed or treated at PHC facilities [13]. This system was first introduced in the Riyadh region in 1986 and was fully implemented across the country by 1992 [14]. A cross-sectional study conducted in Saudi Arabia, involving an online survey administered to 915 patients, revealed that a higher percentage of patients (50.4%) preferred visiting a PHC, whereas 49.6% favored EDs when facing a serious medical condition [15].

In a study conducted across three major hospitals in Al-Qassim Province, namely, KFSH, Buraidah Central Hospital (BCH), and King Saud Hospital, it was revealed that out of 266 patients in the emergency room (ER), a significant 85.7% had prior experience of visiting PHC facilities. The majority of these patients expressed dissatisfaction with their PHC treatment. Specifically, 52.9% of patients cited limited operating hours at PHCs as a concern, 38.1% mentioned a shortage of experienced staff, and 31.7% believed that PHCs lacked adequate diagnostic capabilities. An additional 13.8% of patients reported the unavailability of prescribed medications. On the other hand, 17.7% of the patients reported that they never bypassed PHCs [16].

In a systemic review conducted in 1997, it was observed that after the establishment of the referral system, there was a significant increase in the number of referral cases, with the attendance rate of the ED increasing by approximately 74.1% in the Riyadh region [17]. Furthermore, in a study conducted at the Wasat Abha PHC center in the Asir region, it was found that out of every 100 referrals, 3.7% of patients were referred to the ED [18].

Hence, shedding light on the causes behind non-urgent visits to CTAS IV and V levels of EDs holds the potential to enhance healthcare systems and services. This can lead to time and cost savings while ensuring that ED access is prioritized for urgent cases in need of immediate medical intervention. The primary objective of this study was to evaluate the proportion of non-urgent visits to the ED and identify the factors linked to non-urgent visits in the Al-Qassim region of Saudi Arabia.

## Materials and methods

This study employed a cross-sectional design, utilizing a Google Docs survey (Appendix 1) for data collection from July 15 to December 31, 2022, to capture a snapshot of participant experiences during this period. The total dataset comprised 500 entries, of which 75 were excluded from the analysis due to non-compliance with the inclusion criteria. Thus, the research comprised a sample of 425 patients selected from two prominent healthcare institutions in the Al-Qassim region of the Kingdom of Saudi Arabia, specifically from the Buraidah King Fahad Specialized Hospital and Buraidah Central Hospital. The study encompassed all patients admitted to the ED aged between 20 and 80 years, during the time period from 8:00 to 16:00, coinciding with the availability of PHC centers. The patients incorporated into the study were classified as either less urgent or non-urgent based on the CTAS criteria [19]. Patients under 18 years old, those classified as CTAS 1 or CTAS 2, and patients over 80 years old were excluded from the study.

The choice of these facilities aimed to ensure a diverse representation of patients from the region, providing valuable insights into their perspectives. The data collection instrument comprised two sections: sociodemographics (age, gender, education, and name of facility) and ED questions, primarily using multiple-choice responses, with one open-ended query to allow participants to provide additional context and feedback. Ethical approval (H-04-Q-001) was obtained from the regional research ethics committee at the National Committee of Bio and Medical Ethics (NCBE), demonstrating a commitment to conducting this study with the highest ethical standards. Data collection strictly adhered to these guidelines, preserving anonymity, and protecting participants' privacy with informed consent. Data analysis was performed using R [20] script language version 4.2.3 (R Core Team, https://www.R-project.org/).

The study analyzed non-urgent cases' prevalence in the 2022 ED, using a chi-square test for associations between dependent (urgent/non-urgent cases) and independent variables (facility, age, education, gender, ED visits, complaint duration, recent ED/PHC visits, admissions, ICU history, triage knowledge, priority, and ED/PHC rates). Significant variables underwent logistic regression, presenting results as adjusted odds ratios (ORs) with 95% confidence intervals (CIs). Findings were visually conveyed through tables and figures.

## Results

Among the individuals who visited the ED in 2022, 350 (82.4%) sought care for non-urgent cases, while 75 (17.6%) sought medical care for urgent cases. The prevalence of non-urgent cases was determined based on the number of ED visits. Among the cohort, 118 individuals (33.7%) had never visited the ED, while 99 (28.3%) had one ED visit, 65 (18.6%) had two visits, and 25 (7.1%) had three visits. Those with more than three ED visits numbered 43 (12.3%). For urgent cases, 28 individuals (37.3%) in the cohort had never visited the ED, while 17 (22.7%) had a single ED visit, 14 (18.7%) had two visits, and eight (10.7%) had three visits. Those with more than three ED visits also numbered 8 (10.7%).

In this study, the dependent variable was the binary categorization of ED visits. The following variables were treated as independent variables: facility type, age, level of education, gender, complaint duration, ED visits within the last 72 hours, prior PHC visits before ED attendance, previous normal admissions, prior ICU admissions, waiting time for physician examination, familiarity with the triage system in the ED, belief in the priority for physician assessment, and ratings for ED and PHC services. These variables were cross-tabulated with the urgency of ED visits to determine significant chi-statistics.

Significant findings were observed in relation to age (*p* < 0.001), level of education (*p* = 0.006), ED visits in the last 72 hours (*p* = 0.024), prior admissions (*p* = 0.002), and the anticipated time for assessment by a physician (*p* = 0.002). These significant variables were subsequently subjected to binary logistic regression analysis. For additional details regarding demographic characteristics and their corresponding frequencies and percentages, please refer to Table 1.

**Table 1:** Demographic characteristics data summary segregated by emergency departments (EDs)' urgent and non-urgent visits. Note: *p < 0.05

Variable	Characteristic	Non-urgent* n* (%)	Urgent *n* (%)	p-value
Facility	Buraidah Central Hospital	177 (41.6%)	33 (7.8%)	0.302
	King Fahad Specialist Hospital	173 (40.7%)	42 (9.9%)	
Age	Less than 20 years	38 (8.9%)	3 (0.7%)	<0.001*
	20-34 years	145 (34.1%)	12 (2.8%)	
	35-49 years	91 (21.4%)	27 (6.4%)	
	50-64 years	44 (10.4%)	22 (5.2%)	
	65-80 years	32 (7.5%)	1 (2.6%)	
Education level	Non-formal education	31 (7.3%)	13 (3.1%)	0.006*
	Primary school	41 (9.6%)	12 (2.8%)	
	Middle school	30 (7.1%)	13 (3.1%)	
	Secondary school	119 (28%)	20 (4.7%)	
	College degree	129 (30.4%)	17 (4%)	
Gender	Female	170 (40%)	28 (6.6%)	0.077
	Male	180 (42.4%)	47 (11.1%)	
ED visit in a year	Never	118 (27.8%)	28 (6.6%)	0.722
	One visit	99 (23.3%)	17 (4%)	
	Two visits	65 (15.3%)	14 (3.3%)	
	Three visits	25 (5.9%)	8 (1.9%)	
	More than three visits	43 (10.1%)	8 (1.9%)	
Complain duration	Less than a day	127 (29.9%)	34 (8%)	0.427
	Less than a week	124 (29.2%)	24 (5.6%)	
	More than a week	54 (12.7%)	11 (2.6%)	
	More than a month	45 (10.6%)	6 (1.4%)	
ED visit last 72 hrs	No	323 (76%)	63 (14.8%)	0.024*
	Yes	27 (6.4%)	12 (2.8%)	
PHC visit prior ED visit	No	272 (64%)	58 (13.6%)	0.943
	Yes	78 (18.4%)	17 (4%)	
Admission done before	No	215 (50.6%)	60 (14.1%)	0.002*
	Yes	135 (31.8%)	15 (3.5%)	
Previous ICU admission	No	325 (76.5%)	71 (16.7%)	0.573
	Yes	25 (5.9%)	4 (13.8%)	
Time expected to be seen	Right now,	50 (11.8%)	0 (0%)	0.002*
	Within 10 minutes	57 (13.4%)	10 (2.4%)	
	Within 30 minutes	103 (24.2%)	22 (5.2%)	
	More than 30 minutes	140 (32.9%)	43 (10.1%)	
Knowledge on the ED triage system	I do not know	146 (34.4%)	40 (9.4%)	0.094
	Weak	53 (12.5%)	11 (2.6%)	
	Medium	98 (23.1%)	20 (4.7%)	
	Excellent	53 (12.5%)	4 (0.9%)	
Priority to be seen	No	211 (49.6%)	39 (9.2%)	0.186
	Yes	139 (32.7%)	36 (8.5%)	
ED rate	1	20 (4.7%)	6 (1.4%)	0.235
	2	25 (5.9%)	8 (1.9%)	
	3	99 (23.3%)	21 (4.9%)	
	4	97 (22.8%)	12 (2.8%)	
	5	109 (25.6%)	28 (6.6%)	
PHC rate	1	32 (7.5%)	9 (2.1%)	0.338
	2	60 (14.1%)	6 (1.4%)	
	3	87 (20.5%)	18 (4.2%)	
	4	72 (16.9%)	17 (4%)	
	5	99 (23.3%)	25 (5.9%)	

The second objective aimed to investigate potential associations between patient gender and age preferences and non-urgent ED visits in the Al-Qassim region of Saudi Arabia. The chi-statistics findings noted significant differences between non-urgent and urgent ED visits among patients aged between 20 and 34 years and those aged between 50 and 64 years. However, the findings regarding gender were non-significant (Table 2).

**Table 2:** Chi-statistics based on gender and age preferences on non-urgent emergency department visits. Note: *p < 0.05

Variable	Characteristic	Non-urgent *n* (%)	Urgent *n* (%)	p-value
Age	Less than 20 years	38 (10.9%)	3 (4%)	0.074
	20-34 years	145 (41.4%)	12 (16%)	0.0008*
	35-49 years	91 (26%)	27 (36%)	0.204
	50-64 years	44 (12.6%)	22 (29.3%)	0.001*
	65-80 years	32 (9.1%)	11 (14.7%)	0.251
Gender	Male	180 (51.4%)	28 (37.3%)	0.134
	Female	170 (48.6%)	47 (62.7%)	0.181

Table 3 reveals no statistically significant relationship between the age and gender of patients with medical conditions categorized as urgent ED visits. However, among patients who visited EDs with medical conditions categorized as non-urgent, there were statistically significant negative associations among patients aged less than 20 years (adjOR = 0.207, 95% CI: 0.060-0.711), those aged 20-34 years (adjOR = 0.222, 95% CI: 0.074-0.669), and those aged 35-49 years (adjOR = 0.207, 95% CI: 0.062-0.695), with patients aged above 65 years serving as the reference group. No significant associations were observed among patients aged 50-64 years.

**Table 3:** Patient gender and age preferences for non-urgent emergency department visits Notes: *p < 0.05; AdjOR = adjusted odds ratio, CI = confidence interval

	Dependent: ED visits					
Independent	Non-urgent	CI	*p*-value	Urgent	CI	*p*-value
Age group (less than 20 years)	0.207**	0.060-0.711	0.012	0.246	0.016-3.796	0.315
Age group (20-34 years)	0.222**	0.074-0.669	0.007	0.477	0.086-2.648	0.397
Age group (35-49 years)	0.385	0.122-1.215	0.104	1.02	0.234-4.448	0.979
Age group (50-64 years)	0.207**	0.062-0.695	0.011	2.018	0.408-9.978	0.389
What is your gender (Male)	1.412	0.892-2.237	0.141	1.807	0.620-5.263	0.278
Constant	6.02		0.001	1.354		0.652

**Figure 1:**
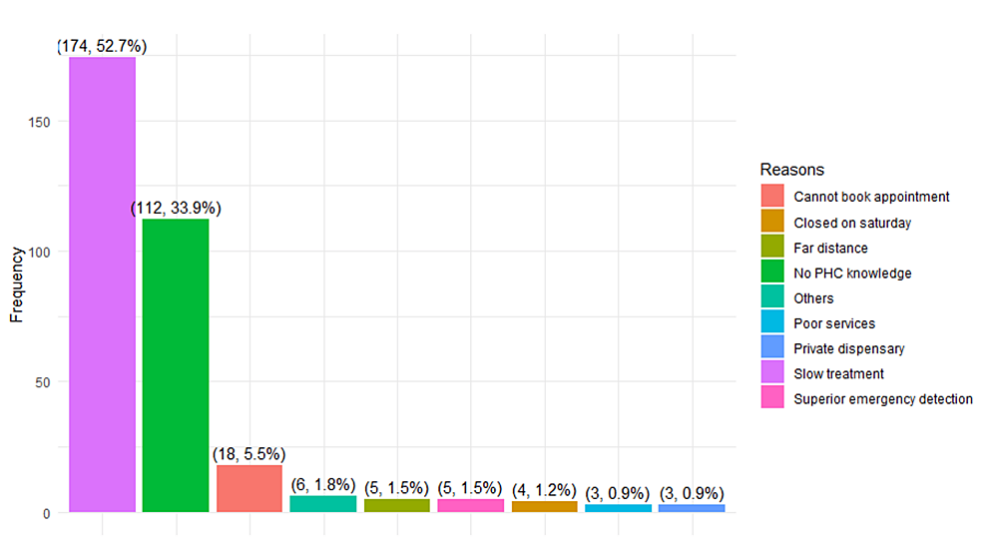
Factors influencing patients' decision to forego seeking primary healthcare (PHC) services before visiting the emergency department (ED).

**Figure 2:**
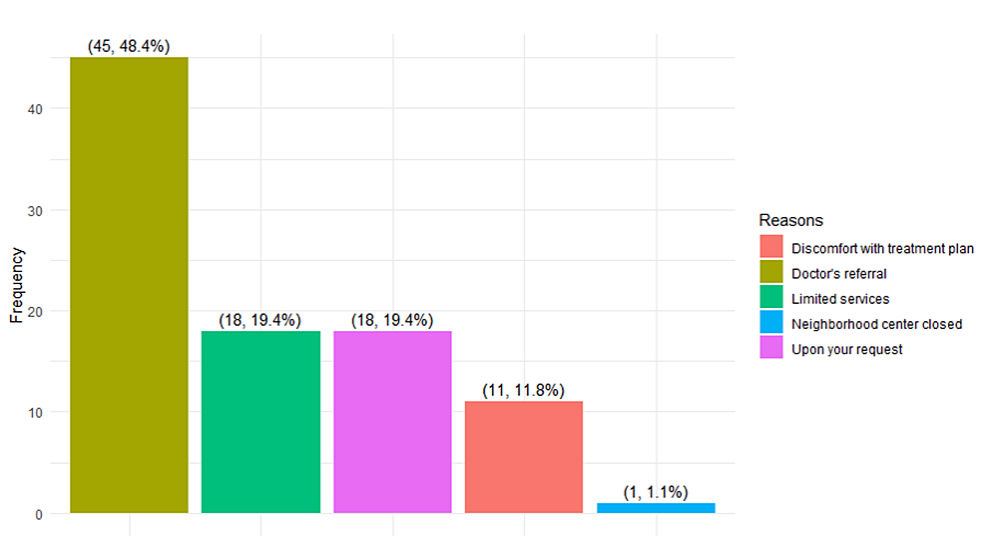
Factors influencing the decision to seek emergency department (ED) services after visiting the primary healthcare (PHC) center.

The third objective assessed several factors contributing to non-urgent cases visiting the ED. A comprehensive review of various factors, including those derived from independent variables and qualitative open-ended questions, was conducted. As shown in Figure 1, it is evident that the top three reasons influencing patients' decisions to forgo seeking PHC services before visiting the ED were slow treatment (52.7%), lack of knowledge about PHC services (33.9%), and the inability to secure an appointment (5.5%). Likewise, patients provided reasons for their visits to the ED after initially seeking care at PHC centers. The majority (48.4%) mentioned visiting the ED upon their doctor's referral, while 19.4% cited limited services at the PHC as a contributing factor. In addition, 19.4% stated that they visited the ED at the recommendation of the ED doctor, 11.8% attributed their choice to discomfort with the treatment plan at the PHC, and a small fraction (1.1%) mentioned the nearest PHC center being closed as the last reason for their decision (Figure 2).

**Figure 3:**
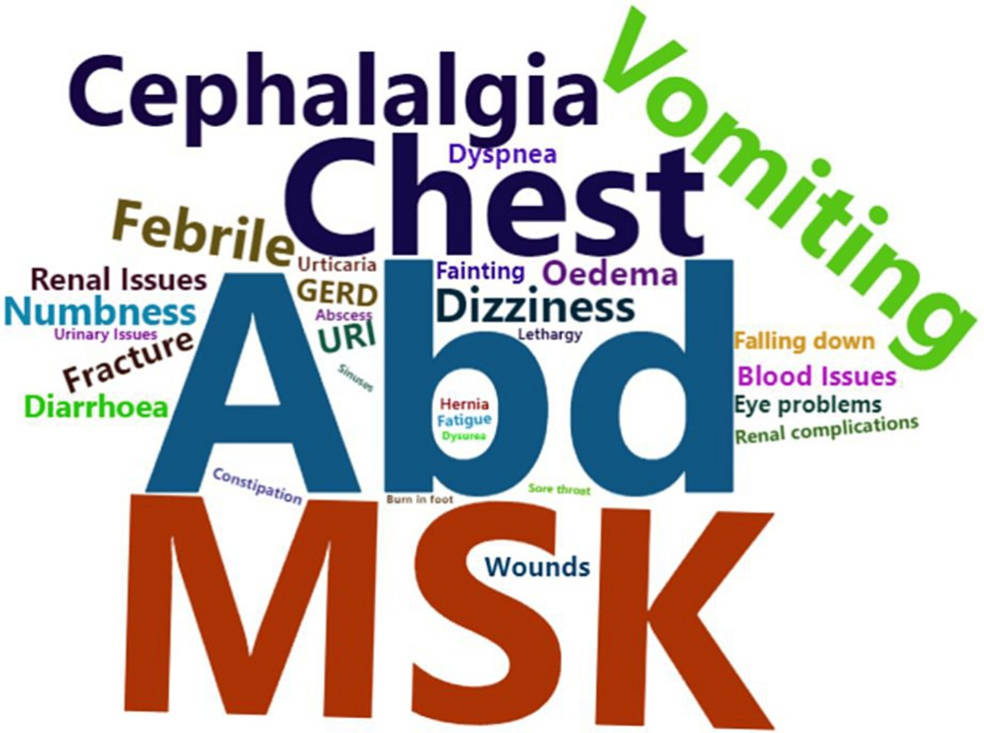
The word cloud displaying the most frequently recorded cases at the ED in the Al-Qassim region of Saudi Arabia.

Various health conditions were reported by patients in response to an open-ended question regarding their reasons for seeking medical services at the ED. These health conditions were documented, and a word cloud was created to visually represent the 32 most common health conditions, as depicted in Figure 3. The specific percentages of health conditions are presented in table format in Appendix 2. The word cloud was generated using the "wordcloud2" package [21] in the R programming language. The word cloud included all conditions that had a minimum of 1% occurrence. In the word cloud, the size of each health condition corresponds to its percentage of occurrence; larger sizes represent higher percentages, and vice versa.

Logistic regression was performed using the following independent variables, which showed significant associations as previously demonstrated in Table 1: the patient's ED visit in the last 72 hours with the same condition, whether the patient had been previously admitted to the hospital, and the expected appointment time with the doctor.

The logistic regression, as presented in Table 4, indicates that an ED visit in the last 72 hours had a statistically significant negative association with patients whose medical conditions were categorized as non-urgent visits at the ED (adjOR = 0.067, 95% CI: 0.009-0.501) and those whose medical conditions were categorized as urgent (adjOR = 0.103, 95% CI: 0.012-0.873), with those who had never visited the ED serving as the reference group. No significant associations were reported for admission and appointment by the doctor.

**Table 4:** Contributing factors for non-urgent emergency department visits Notes: *p < 0.05; AdjOR = adjusted odds ratio; CI = confidence interval

	Dependent: ED visits					
Independent	Non-urgent	CI	*p*-value	Urgent	CI	*p*-value
ED visit in last 72 hours (Yes)	0.067**	0.009-0.501	0.009	0.103**	0.012-0.873	0.037
Admitted before (Yes)	1.222	0.766-1.948	0.4	0.618	0.181-2.112	0.442
Appointment time (Right now)	0.699	0.356-1.374	0.299	0.486	0.109-2.163	0.344
Appointment time (Within 10 minutes)	0.864	0.445-1.679	0.667	0.932	0.305-2.845	0.902
Appointment time (Within 30 minutes)	1.313	0.744-2.316	0.347			
Constant	23.37		0.003	21.09		0.015

The fourth objective centered on evaluating patients' awareness of the triage system within the ED. Overall, the chi-statistics for the assessment of awareness regarding the triage system showed a significant difference (*p *= 0.003) only among patients whose awareness of the ED triage system was excellent. When the findings were stratified by facility, a significant difference (*p *= 0.008) was observed only among patients who had visited KFSH and had excellent awareness of the ED triage system (Table 5).

**Table 5:** Assessment of patient awareness regarding the ED triage system. *Note:* *p < 0.05

Variable	Characteristic	Non-urgent *n *(%)	Urgent *n* (%)	p-value
Overall awareness score	I do not know	146 (41.7%)	40 (53.3%)	0.233
	Weak	53 (15.1%)	11 (14.7%)	0.944
	Medium	98 (28%)	20 (26.7%)	0.860
	Excellent	53 (15.1%)	4 (5.3%)	0.003*
Buraidah Central Hospital	I do not know	85 (48%)	21 (63.6%)	0.140
	Weak	30 (16.9%)	5 (15.2%)	0.924
	Medium	39 (22%)	5 (15.2%)	0.265
	Excellent	23 (13%)	2 (6.1%)	0.115
King Fahad Specialist Hospital	I do not know	61 (35.3%)	19 (45.2%)	0.269
	Weak	23 (13.3%)	6 (14.3%)	0.850
	Medium	59 (34.1%)	15 (35.7%)	0.848
	Excellent	30 (17.3%)	2 (4.8%)	0.008*

The patient's level of awareness of the ED triage system was regressed against the urgency of medical services. Significant associations were observed only among patients who had no prior knowledge of the ED triage system at KFSH, patients who acknowledged visiting the ED within the past 72 hours for the same condition, patients with no previous admission records, those who had not been previously admitted to the ICU, and those who expected to be seen by the doctor at the ED in more than 30 minutes (Table 6).

**Table 6:** Awareness of the ED triage system Notes: *p < 0.05; OR = odds ratio; CI = confidence intervals

		Dependent: ED visits		
	Awareness	OR	CI	p-value
(1) Knowledge of the ED triage system	I do not know	3.630	1.239-10.634	0.019*
	Weak	2.750	0.823-9.186	0.100
	Medium	2.704	0.878-8.324	0.083
(2) King Fahad Specialists Hospital	I do not know	4.672	1.021-21.39	0.047*
(3) ED visit 72 hours	I do not know	3.259	1.101-9.647	0.033*
(4) No Admission before	I do not know	3.923	1.123-13.706	0.032*
(5) No ICU Admission before	I do not know	3.964	1.152-13.642	0.029*
(6) Appointment time (>30 minutes)	I do not know	5.238	1.156-23.745	0.032*

In Table 1, it is evident that most patients with non-urgent medical conditions gave a maximum rating of 5 to both ED services (25.6%) and PHC services (23.3%). The logistic regression analysis of patient satisfaction, assessed on a 1 to 5 scale for ED and PHC experiences (Table 7), revealed significant results among patients aged 20-34 years (*p *= 0.036) and males (*p *= 0.025).

**Table 7:** Significant satisfaction levels with the emergency department (ED) and primary healthcare (PHC) services. Note: *p < 0.05

Variable	Characteristic	Satisfaction level	OR	CI	p-value
Age	20-34 years	PHC level 2	0.293	0.093-0.922	0.036*
Gender	Male	ED level 3	2.722	1.134-6.538	0.025*

## Discussion

This study examined non-urgent visits to ED in the Al-Qassim region of Saudi Arabia from July to December 2022. The participants were selected from Buraidah Central Hospital and KFSH. The investigation aimed to shed light on healthcare utilization patterns, demographics, motivations, and awareness of the triage system, offering insights to optimize healthcare delivery.

The prevalence of non-urgent cases visiting both Buraidah Central Hospital and KFSH in Al-Qassim amounted to 82.4%, with a higher occurrence among females (85.9%) than males (79.3%). This prevalence slightly exceeded the 78.5% reported in Al-Otmy et al.’s [9] study in Saudi Arabia and the 61.4% from Alnasser et al.'s [22] study on non-urgent cases. While the differences were not significant, the prevalence of seeking medical services in the ED at Buraidah Central Hospital was higher (84.3%) compared to the 80.5% reported for KFSH.

The study observed that individuals with a higher level of education were the largest group seeking ED services for non-urgent cases, in contrast to patients with non-formal education (70.5%), patients with primary education (77.4%), and those with a middle school level of education, which was recorded at 69.8%. The current study found a statistically significant association between the level of education, whereas Al-Otmy et al.'s [9] study did not report any significant association.

The study also discovered that the majority of individuals seeking non-urgent medical services in the ED were under 20 years old (92.7%) and aged 20-34 (92.4%). By contrast, a lower percentage of older patients sought non-urgent ED services, with 66.7% in the 50-64 age group and 74.4% in the 65-80 age group. These results contradicted Al-Otmy et al.'s findings [9], which showed a higher proportion of urgent visits among older patients. However, both studies reported statistically significant associations with age.

In Al-Qassim, 22.4% of the patients attempted to visit a PHC before coming to the ED, while 77.6% came directly to the ED (300 patients). This finding contrasts with Almulhim et al.'s [15] study, which reported that the majority of patients (50.4%) preferred visiting PHCs, using EDs only when they had serious medical conditions (49.5%). This pattern of not seeking PHC services before ED services was most prevalent among patients at Buraidah Central Hospital, with 84.8% (178 patients), compared to those at KFSH, with 70.7% (152 patients). These findings are consistent with the findings in Al-Otmy et al.'s study [9], which revealed that despite the availability of several PHCs, most Saudis continued to visit EDs for non-urgent conditions.

In the analysis of non-urgent medical cases, age displayed significant negative associations. Patients under 20 had significantly lower non-urgent case odds (adjOR 0.207), as did those aged 20-34 (adjOR 0.222) and 35-49 (adjOR 0.207). However, no significant associations were observed for patients aged 50-64, indicating similar non-urgent case likelihood to those over 65. These findings emphasize the significant influence of age on non-urgent cases in ED visits, showing that younger age groups are less likely to have non-urgent cases compared to those aged 65 and older. This aligns with Al-Otmy et al.'s [9] study and Alnasser et al.'s [22], which reported that older patients were more likely to have non-urgent visits, contrasting with Khan et al.'s [8], which found that younger patients were the majority among non-urgent medical cases in the ED.

By contrast, no statistically significant gender-based associations were found with non-urgent medical cases, suggesting that gender does not significantly affect the differentiation between urgent and non-urgent cases in the ED. Similar findings were reported by Al-Otmy et al. [9], who found no significant gender differences in patients seeking ED medical services for non-urgent or urgent cases. However, it is important to note that there were minimal differences in the percentages of females (40%) and males (42.4%) visiting the EDs in the current study for non-urgent medical conditions. These results align with Hooker et al.'s [23] study but contradict studies conducted in Saudi Arabia, such as those by Al-Otmy et al. [9], who reported 55% females; Zakaria et al. [24], who reported 57% females; and Al-Surimi et al. [25], who reported 53% females.

Concerning the annual number of ED visits, the current study found no statistically significant difference between patients with non-urgent and urgent medical cases, in line with the results reported by Al-Otmy et al. [9]. It is worth noting that the frequency of ED visits decreased, ranging from one visit (23.3%) to three visits (5.9%).

The study's qualitative findings revealed several factors influencing patients' choice to visit Buraidah Central Hospital and KFSH instead of seeking PHC services. These factors included slow treatment, limited knowledge about PHC facilities, inability to schedule appointments at PHCs, distance from their residence to the PHC, lack of advanced emergency detection at PHCs, weekend closures at PHC facilities on Saturdays, subpar healthcare services, and the perception of PHC facilities as private dispensaries. Similar sentiments were expressed by patients in studies [9,11,26-28], citing challenges in booking appointments, ease of access, the belief that their medical condition was urgent, limited services at PHCs, and referrals from PHCs as the primary reasons for choosing ED over PHCs. Similarly, the study observed that factors, such as doctors' referrals [29], limited PHC services, treatment plan discomfort at PHCs upon doctors' request, and PHC facility closures, influenced the decision to seek ED services after visiting PHCs.

The study's word cloud analysis revealed that abdominal pain was the most prevalent condition, followed by musculoskeletal pain, chest pain, vomiting, nausea, headache (cephalalgia), dizziness, fever symptoms, numbness, fractures, and upper respiratory infections, making up the top 10 health conditions. These findings were consistent with those of Idil et al. [11], which identified muscular pain and upper respiratory tract symptoms as the most common complaints among urgent cases. Similarly, Alnasser et al. reported upper respiratory tract infections as a common reason, and Carret et al. [30] cited respiratory problems, abdominal pain, and chest pain as common causes for non-urgent ED visits.

The logistic regression results show a significant inverse correlation between recent ED visits (within 72 hours) and non-urgent cases, indicating that these patients were 0.067 times less likely to have non-urgent conditions, even when other variables were considered. Likewise, the findings reveal a similar negative relationship for urgent cases, with those who had an ED visit within the last 72 hours being 0.103 times less likely to have urgent conditions, even after adjusting for other factors. This study reports that recent ED visits are linked to a decreased likelihood of both non-urgent and urgent medical cases, suggesting that patients who visited the ED within the past 72 hours are less likely to have either condition.

No significant associations were found regarding factors like admissions and doctor's appointments. In this study, 35.3% of patients documented hospital admissions, with 38.6% classified as non-urgent cases and 20% as urgent cases. This admission rate was higher than the 20.3% reported in Al-Otmy et al.'s [9] study, although 59.3% represented non-urgent cases. Buraidah Central Hospital had a higher rate of hospital admissions among non-urgent cases (42.9%) compared to KFSH (34.1%). Similarly, Buraidah Central Hospital reported 27.3% admissions among urgent cases compared to 14.3% from KFSH.

Regarding awareness levels, a considerable difference was noted among parents with excellent awareness. This was particularly evident in patients at KFSH, where 17.3% had non-urgent cases and 4.8% had urgent cases. This indicates that a significant portion of patients had a high level of awareness about the ED triage system but chose to visit KFSH for non-urgent cases instead of PHCs. After re-coding ED visits as a binary variable, the current study found a substantial percentage of ED visits, with 66.3% for non-urgent medical cases. By contrast, only 22.7% of patients opted for PHCs for non-urgent cases, and the remaining 22.7% had urgent medical cases. The high proportion in this study, while not directly comparable to Al-Otmy et al.’s [9] findings, reflects a lower level of ED awareness among patients in Saudi Arabia.

Additional logistic analysis revealed a significant increase in unnecessary ED visits, primarily linked to a lack of awareness about the ED triage system. This is supported by statistically significant associations found among patients who had no prior knowledge of the ED triage system at KFSH, those who visited the ED within the past 72 hours for the same condition, patients with no prior admission records, individuals not previously admitted to the ICU, and those who expected a doctor's consultation time of over 30 minutes at the ED. The logit regression results for awareness and satisfaction with PHC services revealed that individuals aged 20 to 34 were statistically less likely to visit the ED compared to the reference group when their satisfaction score for PHC services was 2 on a 1-5 scale. Likewise, the study reported that males were more likely to visit the ED than females when their average satisfaction level for ED services was 3 on the 1-5 scale. These findings align with Nawaf et al.'s [29] study, which showed that patient satisfaction is directly related to their knowledge of triage.

Recommendations

Most complaints in the current study can be diagnosed and treated at PHCs, emphasizing the need for the appropriate use of ED services at Buraidah Central Hospital and KFSH. Raising awareness and educating the Al-Qassim population can alleviate the burden on these hospitals, reduce staff fatigue from treatable medical conditions, save time, and conserve resources for urgent cases. Achieving this goal involves promoting triage knowledge through continuous education in venues, such as mosques, social gatherings, and social media.

Limitations

The study cannot generalize the findings to the entire region of Buraidah because it exclusively focused on two hospitals - KFSH and Buraidah Central Hospital. The Al-Qassim region has two more private hospitals that were not included in the study. If data had been collected from the private facilities, the study could have come up with comparative findings between the public and private hospitals on matters related to the ER.

Another limitation was that individuals above 80 years of age were excluded from the study because the researchers discovered that they had limited communication skills, making it difficult for them to understand what the researchers were asking. The study also excluded patients below 18 years old due to the limited time available for the study caused by overcrowding in the ER. That is why not every patient was included in the study.

## Conclusions

The study comprehensively examined the patterns, demographics, motivations, and triage system awareness at Buraidah Central Hospital and KFSH in Saudi Arabia's Al-Qassim region. The investigation revealed a noteworthy predominance of non-urgent cases among patients seeking care at both facilities, with a higher prevalence among females than males. In addition, it was observed that younger age groups were significantly less inclined to present with non-urgent cases compared to their older counterparts. The primary reasons for selecting the ED over PHCs included challenges in booking appointments, ease of access, the perception of the medical condition as urgent, limited services at PHCs, and referrals from PHCs. Among patients seeking ED services for non-urgent cases at Buraidah Central Hospital and KFSH, the top 10 health conditions reported were abdominal pain, musculoskeletal pain, chest pain, vomiting, nausea, cephalalgia, dizziness, fever symptoms, numbness, fractures, and upper respiratory infections. Furthermore, the findings highlighted the significance of awareness of the ED triage system and satisfaction with PHC services. Younger age groups were less likely to visit the ED compared to older individuals when their satisfaction score for PHC services was 2 on a 1-5 scale. Conversely, males were more likely to visit the ED than females when their average satisfaction level for ED services reached 3 on the 1-5 scale.

In conclusion, this research provides valuable insights into the dynamics of ED utilization and emphasizes the importance of patient awareness, satisfaction, and age-related factors in seeking non-urgent medical care in the Al-Qassim region. These findings offer a foundation for enhancing healthcare delivery and optimizing resource allocation in this context.

## Appendices

Appendix 1: Questionnaire

Unnecessary visits to the emergency departments in the Al-Qassim region

1.  I understand the process and I agree to participate in this study.

a)      Yes

b)      No

2.  Hospital name

a)      Buridah Central Hospital

b)      King Fahad Specialist Hospital

3.  Age group

a)      Less than 20 years old

b)      20-34

c)      35-49

d)      50-64

e)      65-80

f)       More than 80 years old.

4. Education level

a)      Bachelor's degree

b)      High school degree

c)      Middle school degree

d)      Primary school degree

e)      None

5. Gender

a)      Male

b)      Female

6. Why did you come to the ER today?

7. Chiecomplaintin duration

a)      Less than a day

b)      Less than a week

c)      More than a week

d)      More than a month

8. How many times did you visit the emergency department this year? (current visit not included)

a)      Never

b)      Once

c)      Twice

d)      Three times

e)      More than three times

9. Any visit done to the emergency department in the last 72 hours to the same condition?

c)      Yes

d)      No

10. Did you try to visit primary health care before you came?

a)      Yes

b)      No

11. If no, what is the reason that made you not visit primary health care?

a)      Lack of resources.

b)      Faster treatment

c)      I couldn’t find an appointment in primary health care.

12. if yes, why did you come after going to the primary health care?

a)      Referred from the doctor from primary health care

b)      Personal reason

c)      Lack of resources

d)      Not satisfied with the diagnosis or the treatment plan

13. Any admissions done before?

a)      Yes

b)      No

14. if yes, when was the admission done?

a)      Less than a week

b)      Less than a month

c)      Less than a year

d)      More than a year

15. Any previous ICU admissions?

a)      Yes

b)      No

16. If yes, when was the ICU admission done?

a)      Less than a week

b)      Less than a month

c)      Less than a year

d)      More than a year

17. What time are you expected to be seen by the doctor?

a)      Now

b)      In 10 minutes

c)      In 30 minutes

d)      More than 30 minutes

18. What is your level of knowledge in the emergency department triage system

a)      I do not know

b)      Poor

c)      Medium

d)      Great

19. If you come first, does that mean that you have to be prioritized to be seen?

a)      Yes

b)      No

20. Rate the emergency department service provided for you using the scores below.

a)      1

b)      2

c)      3

d)      4

e)      5

21. Rate your experience in primary health care experience using the scores below.

a)      1

b)      2

c)      3

d)      4

e)      5

NB: The public is allowed to re-use the questionnaire and customize it to their needs.

Appendix 2

**Table 8:** Medical conditions reported at the emergency department (ED) in the study among 466 documented health conditions.

RowNumber	Response	Frequency	Percent
1	Abdominal pain	80	17.17
2	Musculoskeletal	74	15.88
3	Chest pain	42	9.01
4	Vomiting	28	6.01
5	Cephalalgia	25	5.36
7	Dizziness	12	2.58
8	Febrile	11	2.36
9	Fracture	9	1.93
10	URI	9	1.93
11	Edema	9	1.93
12	Renal issues	8	1.72
13	Diarrhea	8	1.72
14	Numbness	8	1.72
15	Wounds	7	1.5
16	Blood issues	7	1.5
17	GERD	7	1.5
28	Dyspnea	7	1.5
18	Eye problems	6	1.29
19	Falling down	6	1.29
20	Renal complications	5	1.07
21	Muscular spasm and dystrophy	5	1.07
22	Hernia	4	0.86
23	Urinary issues	4	0.86
24	Escort	4	0.86
25	Fainting	4	0.86
26	Fatigue	4	0.86
27	Lethargy	4	0.86
29	Constipation	4	0.86
30	Abscess	4	0.86
34	Urticaria	4	0.86
31	Burn in foot	3	0.64
32	Sinuses	3	0.64
33	Sore throat	3	0.64
36	Dysurea	3	0.64
37	Gall bladder	2	0.43
38	Diabetes	2	0.43
39	Epistaxis	2	0.43
40	Hematuria	2	0.43
41	Haemorrhoids	2	0.43
42	Regurgitation	2	0.43
43	Seizure	2	0.43
44	Stroke	2	0.43
45	Unconsciousness	2	0.43
46	Tingling sensation	2	0.43
47	Liver tumor	1	0.21
48	Loss of appetite	1	0.21
49	Lower limb weakness	1	0.21
50	Melena	1	0.21
51	Mute	1	0.21
52	Neck stiffness	1	0.21
53	Persistent coughing	1	0.21
54	Prostate	1	0.21
55	Red eye	1	0.21
56	Skin allergy	1	0.21
57	Speach problems	1	0.21
58	Tachycardia	1	0.21
60	Acidosis	1	0.21
62	Breast lump or mass	1	0.21
63	Cat bite	1	0.21
64	Cellulitis	1	0.21
65	Companion for my mother	1	0.21
66	Covid	1	0.21
67	Finger stroke	1	0.21
68	Follow-up after RTA	1	0.21
69	Folly’s catheter change	1	0.21
70	Food poisoning	1	0.21
71	Heartburn	1	0.21
72	HTN	1	0.21
73	Hyperglycemia	1	0.21

## References

[1] GOV.SA. Emirate of Al-Qasim Province Buraidah (2023). GOV.SA. Emirate of Al-Qasim Province Buraidah. https://www.my.gov.sa/wps/portal/snp/agencies/agencyDetails/AC006/!ut/p/z0/04_Sj9CPykssy0xPLMnMz0vMAfIjo8zivQIsTAwdDQz9LQwNzQwCnS0tXPwMvYwNDAz0g1Pz9L30o_ArAppiVOTr7JuuH1WQWJKhm5mXlq8f4ehsYGCmX5DtHg4A0RLY_Q!!/.

[2] (2023). Ministry of Health. Awareness Kingdom of Saudi. https://www.moh.gov.sa/en/awarenessplateform/Pages/default.aspx.

[3] King Fahd Specialist Hospital (2023). King Fahd Specialist Hospital. http://Availablefrom:https://www.kfshb.med.sa.

[4] Born K, Laupacis A (2023). The risks of emergency department overcrowding: Healthy Debate. https://healthydebate.ca/2011/07/topic/politics-of-health-care/ed-wait-times/.

[5] El-Masri M, Bornais J, Omar A, Crawley J (2020). Predictors of nonurgent emergency visits at a midsize community-based hospital system: secondary analysis of administrative health care data. J Emerg Nurs.

[6] Backman AS, Blomqvist P, Lagerlund M, Carlsson-Holm E, Adami J (2008). Characteristics of non-urgent patients. Cross-sectional study of emergency department and primary care patients. Scand J Prim Health Care.

[7] Uscher-Pines L, Pines J, Kellermann A, Mehrotra A (2013). Emergency department visits for nonurgent conditions: systematic literature review. Am J Manag Care.

[8] Khan Y, Glazier RH, Moineddin R, Schull MJ (2011). A population-based study of the association between socioeconomic status and emergency department utilization in Ontario, Canada. Acad Emerg Med.

[9] Al-Otmy SS, Abduljabbar AZ, Al-Raddadi RM, Farahat F (2020). Factors associated with non-urgent visits to the emergency department in a tertiary care centre, western Saudi Arabia: cross-sectional study. BMJ Open.

[10] Bahadori M, Mousavi SM, Teymourzadeh E, Ravangard R (2019). Emergency department visits for non-urgent conditions in Iran: a cross-sectional study. BMJ Open.

[11] Idil H, Kilic TY, Toker İ, Dura Turan K, Yesilaras M (2018). Non-urgent adult patients in the emergency department: causes and patient characteristics. Turk J Emerg Med.

[12] (2023). Kingdom of Saudi Arabia. Health Sector Transformation Program Kingdom of Saudi Arabia. https://www.vision2030.gov.sa/en/vision-2030/vrp/health-sector-transformation-program/#:~:text=With%20a%20focus%20on%20improving%20access%20to%20healthcare%2C,meet%20the%20needs%20of%20every%20member%20of%20society.

[13] Al-Mazrou Y, Al-Shehri S, Rao M (1990). Principles and practice of primary health care.

[14] Al-Mazrou YY, Al-Shammari SA, Siddique M, Jarallah JS (1991). A preliminary report on the effect of referral system in four areas of the Kingdom of Saudi Arabia. Ann Saudi Med.

[15] Almulhim N, Almulhim F, Al Gharash A (2021). Preference for visiting emergency department over primary health care center among population in Saudi Arabia. Cureus.

[16] Alqossayir FM, Alkhowailed MS, Alammar AY, Alsaeed AA, Alamri YY, Rasheed Z (2021). Factors associated with patients bypassing primary healthcare centres in Qassim Region, KSA. J Taibah Univ Med Sci.

[17] Alabbasi KH, Kruger E, Tennant M (2022). Strengthening Saudi Arabia's primary health care through an e-referral system: a case study. Clin Pract.

[18] Khattab MS, Abolfotouh MA, Al-Khaldi YM, Khan MY (1999). Studying the referral system in one family practice center in Saudi Arabia. Ann Saudi Med.

[19] Canadian Association of Emergency Physicians (2013). Canadian Triage and Acuity Scale (CTAS) combined adult/paediatric educational program: participant’s manual.

[20] R Core Team (2023). R Core Team R: A language and environment for statistical computing. R version 4.2.3 ed. Vienna, Austria: R Foundation for Statistical Computing. R: A language and environment for statistical computing. R version 4.2.3 ed. Vienna, Austria: R Foundation for Statistical Computing.

[21] (2023). wordcloud2: Create word cloud by 'htmlwidget'. https://github.com/lchiffon/wordcloud2.

[22] Alnasser S, Alharbi M, AAlibrahim A, Aal Ibrahim A, Kentab O, Alassaf W, Aljahany M (2023). Analysis of emergency department use by non-urgent patients and their visit characteristics at an academic center. Int J Gen Med.

[23] Hooker EA, Mallow PJ, Oglesby MM (2019). Characteristics and trends of emergency department visits in the United States (2010-2014). J Emerg Med.

[24] Zakria I, Ala'ddin A, Alnatour MA, Bashir M (2020). Myth versus reality-emergency department revisits within 72 hours, at a tertiary care hospital in Saudi Arabia [PREPRINT]. Res Sq.

[25] Al-Surimi K, Yenugadhati N, Shaheen N, Althagafi M, Alsalamah M (2021). Epidemiology of frequent visits to the emergency department at a tertiary care hospital in Saudi Arabia: rate, visitors’ characteristics, and associated factors. Int J Gen Med.

[26] Afilalo J, Marinovich A, Afilalo M, Colacone A, Léger R, Unger B, Giguère C (2004). Nonurgent emergency department patient characteristics and barriers to primary care. Acad Emerg Med.

[27] O'Brien GM, Stein MD, Zierler S (1997). Use of the ED as a regular source of care: associated factors beyond lack of health insurance. Ann Emerg Med.

[28] Steele S, Anstett D, Milne WK (2008). Rural emergency department use by CTAS IV and V patients. CJEM.

[29] Alhabdan N, Alhusain F, Alharbi A, Alsadhan M, Hakami M, Masuadi E (2019). Exploring emergency department visits: factors influencing individuals' decisions, knowledge of triage systems and waiting times, and experiences during visits to a tertiary hospital in Saudi Arabia. Int J Emerg Med.

[30] Carret ML, Fassa AC, Domingues MR (2009). Inappropriate use of emergency services: a systematic review of prevalence and associated factors. Cad Saude Publica.

